# Supplementary material for: Mapping the Field in Stress, Anxiety, and Postpartum Depression in Mothers of Preterm Infants in Neonatal Intensive Care
Source: Children (Basel). 2021 Aug 25;8(9):730. doi: 10.3390/children8090730 (PMC8468620; doi:10.3390/children8090730)
Supplement: Supplementary file 1 [file children-08-00730-s001.zip › children-1272595-supplementary.pdf]

Supplementary Materials

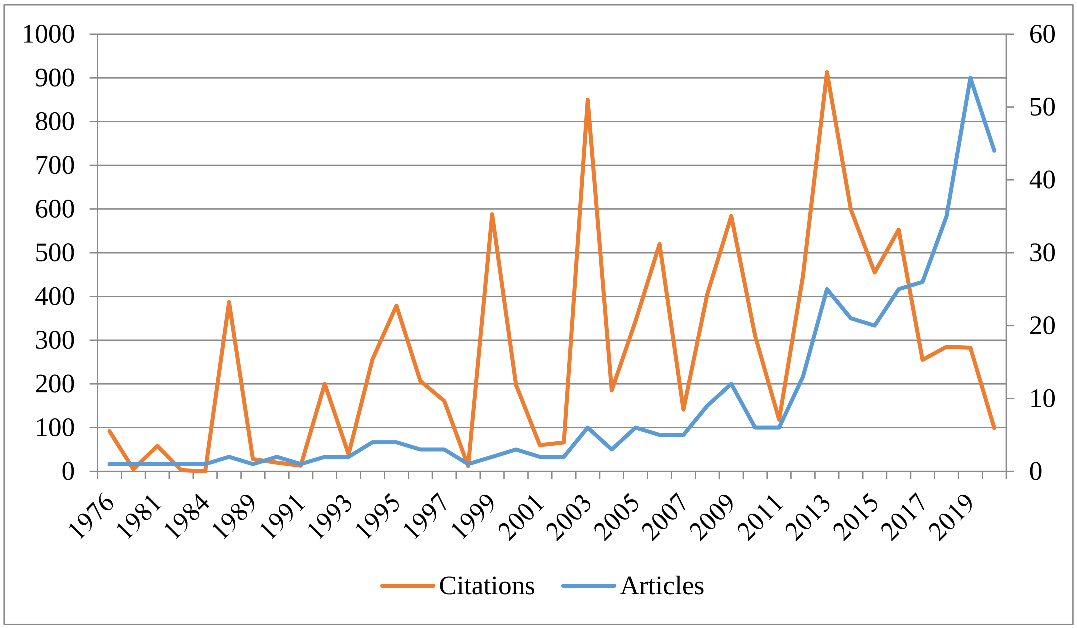

Figure S1. Evolution of published articles and citations from 1976 to 2020. Source: own elaboration based on Scopus 2020.

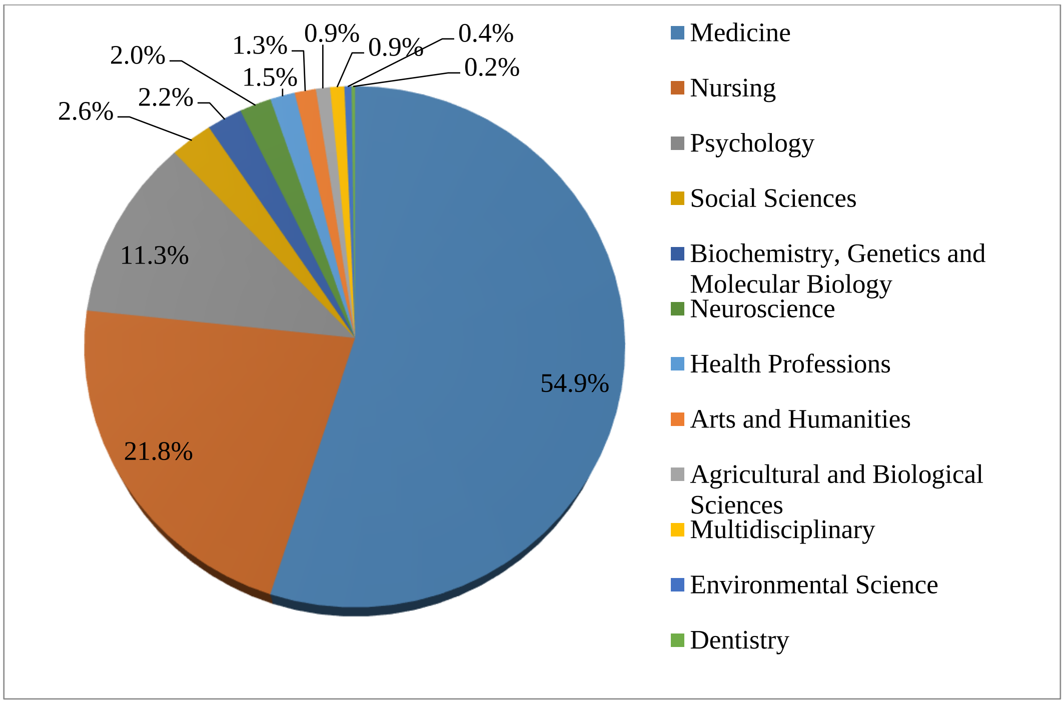

Figure S2. Subject areas that stand out on stress, anxiety and PPD in mothers of preterm infants in NICU 1976 to 2020.Source: own elaboration based on Scopus 2020.

Table S1. The 10 most productive journals in the research on stress, anxiety and postpartum depression in mothers of preterm infants in NICU from 1976 to 2020.

Journal	A	COU	C	C/A	1st A	Last A	C/Y	h-index
Advances In Neonatal Care	17	United States	318	18,71	2005	2020	21.2	10
Early Human Development	17	Netherlands	617	36,29	2003	2020	36.29	11
Journal Of Perinatology	10	United States	533	53,30	1996	2018	24.22	9
Acta Paediatrica International Journal Of Paediatrics	9	Denmark	300	33,33	2008	2020	25	6

Journal Of Clinical Nursing	9	United kingdom	225	25,00	2006	2019	17.30	7
Pediatrics	8	United States	1,145	143,1	1986	2019	34.69	7
BMC Pediatrics	7	United States	113	16,14	2012	2020	14.12	5
JOGNN Journal Of Obstetric Gynecologic And Neonatal Nursing	6	United States	75	12,50	2010	2017	10.71	4
Journal Of Maternal Fetal And Neonatal Medicine	6	United kingdom	129	21,50	2014	2020	21.15	4
Journal Of Perinatal And Neonatal Nursing	6	United States	46	7,67	2014	2019	9.2	3

A: Number of total articles; COU: Countries; C: Number of citations for all articles; C/A: Average citation per article; 1st A: Year of first published article; Last A: Year of last published article; C/Y: Average number of citations per year since the 1st A. Source: own elaboration based on Scopus 2020.

Table S2. The top 10 most productive authors on stress, anxiety and PPD in mothers of preterm infants in NICU from1976 to 2020.

Author	A	C	C/A	1st A	Last A	h-Index	Country	Affiliation
Holditch-Davis, D.	9	634	70,44	1994	2020	7	Unitted States	Duke University, Durham
Miles, M.S.	7	824	117,71	1992	2009	7	Unitted States	University of North Carolina at Chapel Hill
Flacking, R.	6	97	16,17	2013	2019	5	Norway	Oslo University Hospital
Montirosso, R.	6	226	37,67	2012	2018	6	Italy	Scientific Institute IRCCS Eugenio Medea
Shaw, R.J.	6	398	66,33	2009	2014	6	Unitted States	Stanford University
Borgatti, R.	5	134	26,80	2012	2018	5	Italy	Scientific Institute IRCCS Eugenio Medea
Horwitz, S.M.	5	224	44,80	2011	2014	5	Unitted States	University of New Mexico
Martinez, F.E.	5	111	22,20	2004	2016	5	Brazil	Universidade de São Paulo
Mörelius, E.	5	198	39,60	2005	2018	5	Sweden	Linköping University
Anderson, P.J.	4	247	61,75	2010	2019	3	Australia	Monash University, Melbourne

A: Number of total articles; C: Number of citations for all articles; C/A: Average citation per article; 1st A: Year of first published article; Last A: Year of last published article. Source: own elaboration based on Scopus 2020.

Table S3. The top 5 most cited articles from 1976 to 2020.

Title	Author/s	Journal	Year	C	C/Y
Maternal psychological distress and parenting stress after the birth of a very low-birth-weight infant	Singer L.T. Salvator A. Guo S. Collin M. Lilien L. Baley J.	Journal of the American Medical Association	1999	528	25,1
Individualized behavioral and environmental care for the very low birth weight preterm infant at high risk for bronchopulmonary dysplasia: Neonatal intensive care unit and developmental outcome	Als H. Lawhon G. Brown E. Gibes R. Duffy F.H. McAnulty G. Blickman J.G.	Pediatrics	1986	376	11,1
Reducing premature infants' length of stay and improving parents' mental health outcomes with the Creating Opportunities for Parent Empowerment (COPE) Neonatal	Melnyk B.M. Feinstein N.F. Alpert-Gillis L. Fairbanks E. Crean H.F. Sinkin R.A. Stone P.W.	Pediatrics	2006	375	26,8

Intensive Care Unit Program: A randomized, controlled trial	Small L.<br>Tu X.<br>Gross S.J.				
The impact of very premature birth on the psychological health of mothers	Davis L., Edwards H., Mohay H., Wollin J.	Early Human Development	2003	262	15,4
Posttraumatic stress symptoms in mothers of premature infants.	Holditch-Davis D., Bartlett T.R., Blickman A.L., Miles M.S.	Journal of obstetric, gynecologic, and neonatal nursing	2003	226	13,3

C: Total number of citations; C/Y: Average number of citations per year. Source: own elaboration based on Scopus 2020.

Table S4. The characteristics of the strategic diagram topics from 1976 to 2002.

Topics	Documents	h-Index	Citations	Centrality	Density
HUMAN	34	20	2,435	1	1
ADULT	26	16	1, 694	0.92	0.92
INFANT	5	5	1,046	0.58	0.83
ATTITUDE-TO-HEALTH	8	7	514	0.75	0.58
MOTHER-CHILD-RELATION	8	8	339	0.5	0.67
PARENTS	9	7	391	0.67	0.33
ADOLESCENT	7	7	449	0.83	0.17
VERY-LOW-BIRTHWEIGHT	3	3	899	0.25	0.25
CHILD-CARE	2	1	11	0.42	0.75
PREGNANCY	4	4	177	0.33	0.42
INTERVIEW	3	3	689	0.08	0.5
GESTATIONAL-AGE	4	4	345	0.17	0.08

Source: own elaboration based on data from Scopus (2020), generated using SciMAT.

Table S5. The characteristics of the strategic diagram topics from 2003 to 2011.

Topics	Documents	h-Index	Citations	Centrality	Density
FEMALE	52	29	2,936	1	1
RANDOMISED-CONTROLLED-TRIAL	10	10	972	0.73	0.93
CHILD-PARENT-RELATION	13	12	1,186	0.87	0.73
HOSPITALIZATION	13	10	843	0.67	0.8
CHILD-BEHAVIOR	8	7	426	0.53	0.87
INTENSIVE-CARE,-NEONATAL	22	17	1,145	0.93	0.6
YOUNG-ADULT	10	8	444	0.6	0.53
SOCIAL-SUPPORT	9	9	666	0.2	0.33
PRIORITY-JOURNAL	14	11	731	0.47	0.2
PREMATURE-INFANT	7	5	715	0.4	0.4
PSYCHOLOGICAL-ASPECT	20	16	1,208	0.8	0.27
ANXIETY	15	13	952	0.33	0.07
QUESTIONNAIRE	8	7	654	0.27	0.47
NURSING	3	3	176	0.13	0.67
PREGNANCY	3	2	3	0.07	0.13

Source: own elaboration based on data from Scopus (2020), generated using SciMAT.

Table S6. The characteristics of the strategic diagram topics from 2012 to 2020.

Topics	Documents	h-Index	Citations	Centrality	Density
HUMANS	232	32	3,531	1	1
PROSPECTIVE-STUDIES	12	6	236	0.58	0.92
PREGNANCY-OUTCOMES	7	3	91	0.33	0.83
FATHERS	24	13	425	0.83	0.75
BEHAVIOUR	8	6	140	0.42	0.67
VERY-LOW-BIRTHWEIGHT	13	9	200	0.75	0.58
PSYCHOLOGICAL-WELL--BEING	19	9	271	0.92	0.5
PRETERM-BIRTHS	11	5	156	0.67	0.42

VITAL-SIGNS	1	1	5	0.17	0.33
ATTITUDES	2	1	14	0.25	0.25
MOBILE-APPLICATIONS	1	1	9	0.08	0.17
PRETERM-INFANTS	13	6	215	0.5	0.08

Source: own elaboration based on data from Scopus (2020), generated using SciMAT.
